# Imagining Futures: Evaluation of a blended programme of dialectical behaviour therapy and the creative arts for young women with a history of self‐harm

**DOI:** 10.1111/bjc.12528

**Published:** 2025-02-19

**Authors:** L. M. Smith, B. Barrett, S. Barnes, B. Oltean, L. Ige, C. Day, T. Tranah

**Affiliations:** ^1^ Department of Psychology Institute of Psychiatry, Psychology and Neuroscience, King's College London London UK; ^2^ National and Specialist CAMHS, At‐Risk and Forensic Service South London and Maudsley NHS Foundation Trust, Michael Rutter Centre, Maudsley Hospital London UK; ^3^ King's Health Economics King's College London London UK; ^4^ Community Arts North West Manchester UK

**Keywords:** arts, creativity, dialectical behaviour therapy, emotional dysregulation, self‐harm, skills, well‐being

## Abstract

**Objectives:**

To evaluate an arts‐enhanced dialectical behavioural therapy skills group for managing emotions and self‐harm, implemented via an innovative public sector/third sector partnership to increase access to care.

**Design:**

This is a pilot mixed‐methods study.

**Methods:**

To assess participant experience in the ‘Imagining Futures’ programme, we examined self‐report questionnaires and qualitative focus group interviews using framework analysis. We collected recruitment, session attendance and programme completion rates. To explore impact, we also report on quantitative psychological outcome measures, including self‐harm frequency and overall well‐being.

**Results:**

We recruited 45 young women (mean age: 15.9, s.d. = 1.24, range 13.9–18.0 years) with a history of emotional dysregulation, self‐harm and other contextual risks who were not receiving support from statutory child and adolescent mental health services in the United Kingdom. Participants were 22% not in education, employment or training and 77% were from United Kingdom racially minoritized backgrounds. The overall completion rate was 62% (*n* = 28/45). Qualitative data analysed from respondents (*n* = 25/28 young people and *n* = 12 parents) suggested high levels of satisfaction with the project. Thirteen themes were identified which described service elements perceived to support engagement and observed impact. There was an important role for relationships and the creative components. Quantitative clinical data indicated reductions in the frequency of self‐harming, significant reductions in the perceived impact of difficulties and increased perceived social support.

**Conclusions:**

This novel delivery of a DBT skills group, incorporating blended psychology arts activities, has the potential to support engagement with psychological supports that improve mental well‐being.

**Practitioner Points**

- Cross‐sector partnerships might be used to increase reach and access to therapies.
- Dialectical behavioural therapy skills can be flexibly blended with socially engaged arts methods while maintaining an impact on clinical targets.
- Further research is merited into the shared mechanisms of action across arts and psychology programmes.

## INTRODUCTION

Barriers to accessing routine mental health services for young people are well documented and include social stigma, negative beliefs about therapy, high thresholds and long waitlists in statutory provisions (Radez et al., 2021; Velasco et al., 2020). The recent COVID‐19 pandemic has had an overall negative impact on mental health, particularly for young people, and has prompted further scrutiny of the gaps in mental health care provision in the United Kingdom and elsewhere (Chavira et al., 2022; Creswell, 2023; Creswell et al., 2021). Finding solutions to overcome barriers to mental health care remains a complicated task globally (Gulliver et al., 2010). Pre‐pandemic, statutory child and adolescent mental health services (CAMHS) in the United Kingdom were estimated to reach one in four young people in need (Public Health England, 2016; Care Quality Commission, 2017, 2018). Compelling arguments have therefore been made in favour of new models of care, including National Health Service (NHS)/third‐sector partnerships, intended to increase reach and access to therapies for those with intersectional disadvantage (Crenna‐Jennings & Hutchinson, 2020; Newbigging et al., 2020).

Contemporaneously, there has been renewed attention within the health sciences to the role that creative disciplines and infrastructures can play in supporting mental health and well‐being (Baring Foundation, 2020; Fancourt & Finn, 2019). While there is a long tradition of creative therapies, recent reviews have focused on the expanding evidence base for applied, socially engaged arts methods with specific impacts in the prevention and alleviation of mental health issues (All‐Parliamentary Group on Arts, Health and Wellbeing, 2023; Fancourt & Finn, 2019). For example, studies have examined how different participatory arts like acting, singing and dancing, or visual creative arts like drawing or photography, can contribute to improved emotion regulation via specific mechanisms, for example, distraction (diverting attention from a stressor towards other thoughts and behaviours), re‐appraisal (considering alternative interpretations of the meaning of emotion‐eliciting events) or physical modulation (e.g., addressing physical suppression of emotion) (Fancourt & Ali, 2019).

There is increasing evidence to suggest that arts‐based methods are helpful in promoting young people's perceived agency, feelings of empowerment and ability to express more fully their experiences and challenges relating to mental health (Desmond et al., 2015; Fancourt & Finn, 2019). It is often claimed that arts activities provide a more appealing and accessible way to engage with diverse communities and marginalized groups regarding experiences of mental ill health (Pavarini et al., 2021; Zarobe & Bungay, 2017). Additionally, arts methods do not solely prioritize literacy or verbal fluency and may be more acceptable to young people who have experiences of poor attainment, school exclusion or negative experiences with care systems (Crouch et al., 2019; Stempel et al., 2017). The buildings and infrastructures supporting creativity in the community are often more geographically accessible for people and less likely to attract the stigma of mental health or hospital facilities (Anderson et al., 2017; Brown et al., 2016). Nevertheless, it is not a given that arts methods are effective in supporting people's well‐being. They may be perceived as elitist or inaccessible by some (Mason & McCarthy, 2006). Moreover, there are mental health areas which address particular risk issues and concerns in which specific mental health and safeguarding expertise and interdisciplinary working may be necessary.

One such area is that of self‐harming in young people. Rates of self‐harming and attempted suicide among the adolescent population in the UK population have been increasing (McManus et al., 2019; Morgan et al., 2017). Globally, it is estimated that one in five young people has engaged in non‐suicidal self‐injury (Xiao et al., 2022), and a history of self‐harm is one of the strongest predictors of subsequent suicide (McMahon et al., 2022). There is a need for an expansion in efforts to address self‐harming in young people, particularly for those who experience disparities in health care support, and including in geographical areas where unfavourable attitudes towards self‐harming behaviours may have been identified as a key barrier to care programmes (Aggarwal et al., 2021).

Within a local context, while equal attention must be paid to often overlooked groups including young men who self‐harm (Tofthagen et al., 2022), studies in South London have shown that young women, aged between 15 and 19 years have considerably higher rates of hospital admission following self‐harm than other groups (The Nuffield Trust, 2024; Southwark Joint Needs Assessment, 2018). Children who have a history of self‐harming have been shown to be nine times more likely to have died over longitudinal follow‐up studies (with suicide being the main cause of death in young people) (Morgan et al., 2017). Referrals to CAMHS following index episodes of self‐harming are 23% less likely for young patients registered in the most socially deprived areas, even though incidences are considerably higher in these localities (Morgan et al., 2017). Moreover, higher rates of drop out from treatment are reported among ethnic minority young people, suggesting that current provision for various reasons may not meet their needs (Care Quality Commission, 2017; De Haan et al., 2018).

The recently published UK National Institute for Health and Care Excellence (NICE) guidelines on ‘Self‐harm: assessment, management and recurrence prevention’ recommend working together across third sector, educational, social and health services (NICE, September, 2022). Onward referral for specialist assessment of psychosocial risk is recommended for all young people who have self‐harmed. For those who are triaged as requiring further support, evidence‐based cognitive behavioural interventions (CBT) are recommended, including mentalization‐based therapies, and dialectical behavioural therapy (DBT) for adolescents where significant emotional dysregulation is also present. However, there remains debate about how to meet the needs of young people with differing levels of severity of difficulties across different educational and health sectors, including internationally (McManus et al., 2019; Torralba‐Suarez & Olry‐de‐Labry‐Lima, 2024).

DBT is a ‘third‐wave’ CBT which teaches mindfulness skills, alongside techniques for managing emotions, relationships and tolerating distress (Linehan, 1993). The therapy was originally developed for the treatment of highly suicidal adults with symptoms associated with borderline personality disorder (BPD), including instability in emotion regulation, impulse control, interpersonal relationships and self‐harming behaviours (Bohus et al., 2021). The modes of therapy in the comprehensive treatment model include 1–1 clinical sessions, group skills classes and telephone support. The DBT protocols have been adapted for adolescents with emotional dysregulation and self‐harm (Rathus et al., 2018; Rathus & Miller, 2015) and have an established evidence base in treating these behaviours in young people (Kothgassner et al., 2021). The typical length of DBT skills groups in controlled trials of intensive therapy for recurrent self‐harm in adolescents is approximately 20 weeks and is administered with parents/carers adopting a multi‐family approach (McCauley et al., 2018; Mehlum et al., 2014, 2016). However, promising results also exist for the effectiveness of adapted DBT skills interventions of varying lengths, suited to context and service needs (Cook & Gorraiz, 2016; MacPherson et al., 2013). Intensive and standardized DBT is not needed by all, but rather for those with the most severe difficulties (Kothgassner et al., 2020). Yet, DBT remains an attractive framework for adapted and formulation‐led interventions due to its modular and skills‐based approach.

This study therefore sought to address issues of barriers to access to care, increased rates of self‐harm locally and calls for cross‐sector innovations to meet prevention efforts for self‐harming in young people. We integrated established NHS‐led DBT skills groups with community‐led participatory arts activities in a grant‐funded pilot programme for young women with histories of self‐harming. We report here on a mixed‐methods evaluation of this service development, which was also impacted by the COVID‐19 pandemic. We assessed qualitative factors identified as important by participants to their engagement and experience. We examined recruitment methods. We also explored outcomes related to clinical impact, measured at the start and the end of Imagining Futures (IF).

## MATERIALS AND METHODS

### Grant funding

Two of the authors (TT and LMS in collaboration with participatory arts specialist SB) developed a study to evaluate the augmentation of a standardized DBT skills group for young people with multi‐disciplinary arts. The project was supported by a grant from the Guy's and St Thomas's Charitable Foundation.

### Interdisciplinary partnership

The study represents a collaboration between the National and Specialist CAMHS within South London and the Maudsley NHS Foundation Trust (N&S CAMHS, SLaM) and Oval House Theatre—a community arts space in South London. N&S CAMHS provide psychological and psychiatric assessment and intervention for young people and their families/carers across South London and the rest of the United Kingdom. DBT skills groups and adapted 1–1 DBT skills interventions are employed variously across the N&S CAMHS clinical services, alongside other therapies.

### Participants/recruitment

It was a central concern to the project that we widened avenues for referral and the study focused on two boroughs in South London which are known to have high levels of need and deprivation, Lambeth and Southwark. Referrals were accepted directly from young people, carers, local schools, third‐sector youth and arts organizations, social services, youth offending teams and CAMHS. The service was publicized alongside existing N&S services on the SLaM website and via information sheets, videos and posters circulated online and via institutions. Recruitment took place in two 3‐month waves, between February and April 2019, and December 2019 and Mar 2020, for two rounds of the 30‐week programme delivered over consecutive years.

### Inclusion/exclusion criteria

Young women were eligible to join this initial pilot study if they had prior experience (within the last 12 months) of struggling with emotion regulation difficulties and self‐harm (e.g., cutting, scratching, burning or hitting themselves). There were no exclusion criteria related to mental health diagnoses. However, participants had to have a sufficient level of English language and ability to take part in the workshops as designed. This excluded some young women with special educational needs. However, those with neurodevelopment diagnoses, including autism spectrum disorder and attention deficit hyperactivity disorder, who were able to participate were included. The inclusion of women only was informed by the evidence base and a local needs assessment (The Nuffield Trust, 2024; Southwark Joint Needs Assessment, 2018). Participants were accepted if aged between 14 and 17 years old and if they had an appropriate adult to support their referral/application. To ensure we were meeting those young people in need, the intervention was only available to young women who were not in receipt of a targeted mental health intervention elsewhere, for example, via local CAMHS. This included those who had not been offered CAMHS treatments as a result of high thresholds for entry, those who were on long waitlists for CAMHS and those who had disengaged/experienced barriers to accessing therapy, as well as those experiencing gaps in therapeutic offering at the transition from CAMHS to adult services.

### Service evaluation and consent to participate

The aims of this evaluation met criteria for service evaluation rather than research or audit (Twycross & Shorten, 2014; UK Health Research Authority, 2022). It was designed and conducted with the sole purpose of defining or judging the services provided by the At‐Risk and Forensic team within N&S CAMHS. The service evaluation did not explore, or seek to undertake an experiment to investigate or establish broader evidence related to DBT interventions, nor wider research issues related to implementation science. Additionally, a key aspect of the study was ensuring that the design procedures did not present barriers to recruitment and retention of this target population. The study was approved by the SLaM CAMHS Service Evaluation and Audit Committee as an evaluation of service development within the At‐Risk and Forensic Service. Informed consent to participate in the 30‐week skills programme was sought as would be for treatment from young people and a consenting adult where appropriate. All methods were performed in accordance with the SLaM guidelines and regulations, including obtaining consent for audio/video recording of sessions, observation of GDPR, safeguarding and confidentiality policies. Data were anonymized using individual codes. The service received by the young people was not conditional nor affected by taking part in the evaluation.

### Screening process

As part of the process for entry into the programme, young women attended an introductory meeting. This included a 1–1 arts ‘taster’ practice with one of the lead artists, a meeting with a clinical psychologist to complete a screening questionnaire for emotional difficulties and self‐harm and a brief risk assessment. A basic self‐report check list of areas of concern/risk was included in the application form. Individuals were asked to tick if they had experienced a range of difficulties across four categories (emotional health, social environmental, problem behaviours and risk of harm; please see results for further details). Young people were offered a place if they had scores of 4 and above on the McLean Screening Instrument for BPD (Zanarini et al., 2003). In clinical practice, if a client scores 5 or 6, further evaluation for clinical diagnosis is recommended (Zimmerman & Balling, 2021). Individuals were referred to other services if their needs or level of risk required an alternative provision.

### Skills group and blended programme

Within the N&S CAMHS, DBT skills are taught following the standardized modules detailed in the adolescent DBT manuals: mindfulness, distress tolerance, emotion regulation, interpersonal effectiveness and ‘walking the middle path’ (Rathus & Miller, 2002). However, in contrast to the adolescent multi‐family approach, skills classes are typically separated for young people and parents/carers (Smith et al. 2023). To augment existing service approaches, the IF programme allowed for a 50% increase in the length and number of sessions in skills groups to enable creative arts additions. The 30‐week programme was developed in a collaboration between two British Isles' DBT‐trained clinical psychologists in the N&S CAMHS (LMS and TT) and three appointed lead professional artists from Oval House Theatre. A concept mapping exercise was completed which evaluated the principles and skills covered in the DBT adolescent manual (Rathus & Miller, 2015) and sought to identify thematic overlaps with creative arts practices to create integrated session plans. Please see Data S1 and S2 for examples of the final programme and session structure across the three modules, and examples of DBT skills resources developed over the course of the programme. For more information about the programme content, please contact the corresponding authors.

The programme includes the core skills from DBT in distress tolerance, emotion regulation and interpersonal effectiveness, alongside practices to develop specific skills in the professional creative disciplines including drama, photography, music and voice, for example, camera handling, characterization, improvisation, creative writing and voice work. For example, we completed core ‘mindfulness’ exercises during a jewellery‐making session and created ‘self‐soothe’ bags via screen printing methods. Each of the sessions had didactic elements as would exist in standard DBT group skills, with augmentation through the arts practices. The initial programme design was evaluated by a youth steering group (*n* = 6) of individuals who had previously completed DBT treatments with N&S CAMHS, as well as youth workers from Oval House, and their comments were used to adapt elements including the pace and content of sessions and the approach to blending the skills and arts practice.

**FIGURE 1:**
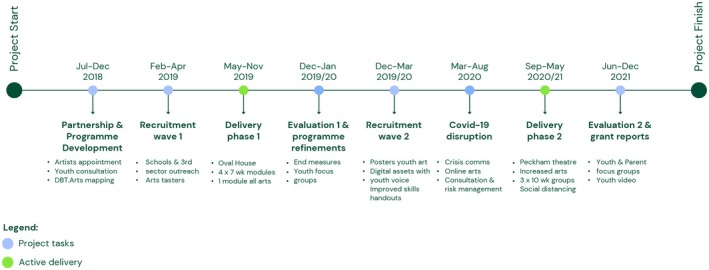
Study Development Phases.

Four modules were run in the first year, with the final module being solely focused on creative activities. In the second year, following feedback, this was consolidated into three longer modules with a ‘creative sharing’ at the end of each module. Additional changes following formative evaluation included diversifying the creative activities, and the provision of improved visual resources informed by young people's artwork. Please see Figure 1 below for the study development phases.

### Training/quality assurance

Each of the sessions in the programme was delivered by a qualified and DBT‐trained clinical psychologist (LMS) working in partnership with the co‐facilitating lead artists. Support for the young people in sessions was provided by an assistant psychologist and an arts youth worker with relevant lived experience and knowledge of DBT skills. In our second year, two volunteer mentors who had been participants in the first year joined the groups. Additional supervision was provided by the project lead (TT) and artistic directors from Oval House. Regular parent/carer ‘drop‐in’ sessions were provided by the project lead and artistic leads to encourage and maintain parent/carer involvement and feedback.

### Procedure and measures

To consider recruitment methods, session attendance rates and programme completion rates were calculated. Attendance data were collected weekly via a register. To obtain quantitative and qualitative information on participant experience, young people completed an adapted version of the Child and Adolescent Service Experience (ChASE) questionnaire (Day et al., 2011). Additionally, qualitative feedback was sought from young people and parents/carers in semi‐structured focus group interviews (Krueger & Casey, 2015). Please see Data S3 for the interview schedules. These were completed in a single session, within 1 week of the end of the programme. For young people, the focus group was conducted in person, in groups of up to seven, facilitated by two interviewers who were not directly involved in the programme delivery. Sessions lasted approximately 25–30 min. For parents and carers, the focus group session was completed online at their request for convenience, with the session lasting approximately 75 min. A proportion of parents/carers responded to interview questions in writing via questionnaires issued online as they were unable to attend the focus group. The interview questions were structured to address reflections relating to the access, engagement, acceptability and impact of the groups.

Routine clinical outcomes were assessed via online questionnaires hosted securely on the SLaM Qualtrics platform and were completed by young people at the start and the end of the programme. In our first year, these were completed together in the first and final sessions with shared tablets. In our second year, young people were distributed links which they completed via personal mobile phones.

Clinical measures included:

- A single question assessing self‐harm frequency (adapted from the *Deliberate Self‐Harm Inventory* (Gratz, 2001)), which asked ‘Over the past 6 months, approximately how many times have you deliberately (on purpose) hurt yourself physically (e.g. punched yourself, cut yourself, burned yourself) without intending to kill yourself’.
- The *Strengths and Difficulties Questionnaire* (Goodman et al., 2000), which is an internationally validated clinical assessment of behavioural difficulties in 2–17 year olds. It assesses emotional and conduct problems, impulsivity and peer relationship behaviour.
- The shortened *Difficulties with Emotion Regulation Scale* (DERS‐18, Victor & Klonsky, 2016), where higher scores indicate greater difficulty with emotion regulation. The DERS‐18 has been validated on large samples of adolescents and has been shown to retain the excellent psychometric properties of the original and widely used long‐form DERS (Gratz & Roemer, 2004; Skutch et al., 2019).
- The *Warwick‐Edinburgh Wellbeing questionnaire* (Stewart‐Brown et al., 2011), which is an accessible 14‐item scale measuring both the feeling and functioning aspects of mental well‐being. It has been used nationally and internationally for monitoring and evaluation of well‐being in the general population.
- The 12‐item *Multidimensional Scale of Perceived Social Support* (Zimet et al., 1988), which is a short measure of the perceived level of social support a person receives from family, friends and significant others.

We also assessed basic socio‐demographic and clinical health service use data via self‐report:

- Self‐identified ethnicity (UK Census Ethnicity Categories)
- Education status
- A measure of clinical health service use (including accident and emergency visits, ambulance call‐outs),
- Hospital appointments (for mental health, injury or other reasons and assessing overnight stays),
- Outpatient health and school appointments, including GP/doctor, nurse/midwife/health visitor, CAMHS practitioner, social worker, school nurse, counsellor/psychologist and youth offending team worker.

Those who opted‐out during the programme and did not complete the sessions were followed up by telephone and invited to complete a structured opt‐out interview exploring reasons for non‐attendance. Participants who completed the opt‐out interviews were offered a £5 voucher reimbursement for their time.

### COVID‐19 adaptations

The second year of the programme was due to commence in March 2020 and was stalled due to the COVID‐19 pandemic. Invitations to participate in arts activities via Instagram were provided to maintain engagement with participants who had already enrolled. For example, personal sketch books were sent by post to design the front covers of these and participants were invited to create Bitmojis of items observable in the rooms that were of significance to them. Advice was sought from the UK Government COVID helpline, the National Youth Agency and Lambeth Council in interpreting guidance for third‐sector groups supporting vulnerable young people in the community. When permitted to resume delivery, 6 months later, sessions and assessment methods were adapted to follow COVID safety guidance regarding social distancing and hand hygiene.

### Analysis plan

Descriptive statistics were used to summarize socio‐demographic information (age, ethnicity and ‘Not in Education, Employment or Training’, NEET and status) as well as clinical health service use data. Comparisons in quantitative clinical data, between start and end of programme, were analysed and reported using paired samples t‐tests. A framework analysis of qualitative responses obtained in the focus groups (total of 5, *n* = 29) and via questionnaire for a subset of parents (*n* = 8) was completed (Gale, 2013), with each focus group interview analysed as one response and parent/carer feedback provided in questionnaire format analysed as one response. Framework analysis was selected to examine the qualitative data collected due to its strengths in working flexibly with case reports from multiple sources and in multi‐disciplinary contexts (Goldsmith, 2021). Responses were analysed using deductive themes related to pre‐determined topics, as well as remaining open to emergent themes. Participants were considered to have completed the programme if they attended each of modules, including at least one‐third of the sessions in each module and the end of programme final event. The distribution of variables was inspected via histograms and the results of Kolmogorov–Smirnoff tests. Wilcoxon rank‐sum tests were completed for non‐parametric data but did not alter the results. We planned on conducting our analyses following intention‐to‐treat (ITT) approaches; however, attrition in the quantitative data obtained at end of treatment for those who opted out during the programme was so high that we could not reasonably expect data imputation methods to address missing data effectively. Moreover, the inherent assumption that data were missing at random was violated. Independent samples t‐tests were used to assess and report mean differences between treatment completers and non‐completers in baseline clinical data, as well as the Chi‐square test for frequency data or Fisher's exact test where cell counts were less than 5. The remainder of the analyses addressing quantitative data were conducted and reported on treatment completers (*n* = 28/45). Confidence intervals of 95% were used.

## RESULTS

### Uptake, attendance and completion

Over the 2 years, a total of 99 applications/referrals were received via the following pathways: 26% schools, 43% CAMHS, 19% Social Services, 3% third sector organizations and 9% direct/family. In year 1, 32 individuals (62% of referrals) were offered a place to begin the workshop programme, with 21 young women enrolling in two separate weekly groups (*n* = 10 and *n* = 11 respectively). The overall completion rate in the first year was 57%, with 65% average session attendance within this completing group. In year 2, a total of 30 individuals (64% of referrals) were offered a place to begin the workshops. Just over half (*n* = 16) of those individuals joined the programme after a delayed start date of 5 months due to COVID‐19 national lockdown procedures. A further 8 young people were recruited in the interim, giving 24 individuals who enrolled in the second year in two cohorts. The overall completion rate in the second year was 67%, with 87% average session attendance. We had two individuals in the second year who started after the first module due to COVID‐19‐related issues, but who were included as programme completers having met the completion criteria in the modules available to them.

### Participants

The mean age of participants (*n* = 45) was 15.9 years (s.d. = 1.24). Mean score on the McLean screening instrument was $\bar{X}$ = 7.82 (s.d. 1.50). Self‐identified ethnicities (*n* = 42) were as follows: Black or Black British (African, Caribbean and other): 34.9%; White British/Irish: 23.3%; mixed White/Black Caribbean: 16.3%; mixed White/Black African: 11.6%; White other: 7.0%; and mixed – other: 7.0%. There was one individual who identified as gender non‐binary, assigned female at birth. One‐fifth of the sample were NEET, although most were in full‐ or part‐time education.

There were no significant differences between completers and non‐completers in age (completers, $\bar{X}$ = 15.95 (s.d. = 1.22); non‐completers, $\bar{X}$ = 15.74 (s.d. = 1.29), *t* = .531, d.f. = 40, *p* = .598; or self‐reported ethnicity, Fisher's exact test, d.f. = 6, *p* = .685). Non‐completers reported statistically higher levels of emotional difficulties than completers at baseline (completers, $\bar{X}$ = 6.78 (s.d. = 2.10); and non‐completers, $\bar{X}$ = 6.87 (s.d. = 1.06, *t* = −.153, d.f. = 40, *p* = .045)). Please see Table 1 in Data S4.

**TABLE 1:** Frequency responses to Likert‐scale items on the adapted ChASE.

	*n*	All of the time	Most of the time	Some of the time	Rarely	Never
Relationship items						
Were the facilitators kind and caring?	23	20 (87%)	3 (13%)	—	—	—
Did you talk about the really important things?	24	4 (17%)	11 (46%)	6 (25%)	3 (12%)	—
Did the facilitators really try to understand you?	23	9 (39%)	9 (39%)	5 (22%)	—	—
Did the facilitators wait until the right moment before asking personal/private stuff?	23	16 (70%)	5 (22%)	1 (4%)	1 (4%)	—
Did the facilitators have good ideas about how to help you?	23	5 (22%)	12 (52%)	6 (26%)	—	—
Did you trust the facilitators?	23	14 (61%)	7 (30%)	2 (9%)	—	—
Did you get the chance to talk about how you were feeling?	24	6 (25%)	8 (33%)	6 (25%)	4 (17%)	—
Privacy Items						
Did you get annoyed or anxious because of any of the questions or activities?	24	2 (8%)	1 (4%)	13 (54%)	5 (21%)	3 (13%)
Was it hard to say things because other young people or adults were there?	24	2 (8%)	6 (25%)	8 (34%)	8 (33%)	—
Were you asked questions that were too personal/private?	23	1 (4%)	1 (4%)	3 (13%)	4 (18%)	14 (61%)
Session activity items						
Were the workshops enjoyable?	24	10 (42%)	14 (58%)			
Did the workshops help you get on with your life?	23	6 (26%)	5 (22%)	10 (43%)	2 (9%)	—
Did you get to do interesting and creative things?	24	11 (46%)	13 (54%)	—	—	—

We had two non‐completers who were being accommodated within residential settings by the local authority as a result of a care order. All of the treatment completers were living with a parent/carer as opposed to being accommodated by the local authority. A comparison in the self‐reported data on mental health and risk experiences from application forms suggested that there were higher rates of bullying, as well as harm from others (in the form of sexual exploitation) in treatment completers, possibly suggesting a role for social support in maintaining attendance (please see Table 2 in Data S4). Also, there were higher rates of active self‐harming in treatment completers, supporting the relevance of the programme. By contrast, non‐completers had higher rates of familial difficulties and an absence of family support which may have impacted attendance and there were also greater behavioural difficulties in this group.

**TABLE 2:** Examples of responses from ChASE open‐response items.

Best things about the group	
Group Values	‘the fact that I never felt judged’ ‘that everyone was super caring and respectful’ ‘I was also very comfortable’ ‘the nurturing environment’
Relationships	‘the facilitators are so nice’ ‘the people (everyone)’ ‘the friends I made’ ‘just being around different people, the workers and the girls’ ‘talking to people in similar situations’ ‘getting to meet new people with similar experiences as you’ ‘other young females’
Variety of Activities	‘I don't know, acting and games’ ‘the different type of activities we did that linked to mental health’ ‘different variety of things to do, it's always something new’ ‘the range of activities, the DBT Skills’ ‘dance session, final show’ ‘games’ ‘new creative activities, interactive activities’
Practicalities	‘the regularity of the sessions’ ‘the food’ ‘having somewhere to go each week’
Impact	‘the facilitators helped me find out who I am’ ‘it is really fun with the people’ ‘able to bring my mood up sometimes’ ‘talking to people, not feeling so alone’

Things to improve in the workshops	
Satisfaction	‘I thought it was good how it was’ ‘nothing’ ‘I can't think of anything it was great’
Activities	‘more hands‐on activities e.g. crafts’ ‘more artistic things’ ‘more focus on the DBT skill’
Practicalities	‘make them longer’ ‘maybe a group for older people’
Communication	‘more opportunities to talk about more pressing issues’ ‘talk about things more than sugar‐coating’ ‘getting to know each other way more’

### Primary outcomes

#### ChASE questionnaire (Day et al., 2011)

Self‐report feedback on the adapted ChASE is shown in Tables 1 and 2 below. Quantitative and open‐ended responses suggested that participants were satisfied with the programme. That most participants rated that they were able to discuss ‘the really important topics’ suggests that it is feasible to consider serious therapeutic issues and skills within an ‘enjoyable’ arts programme. Responses demonstrated that the young people found the programme facilitators to be trustworthy, caring and helpful; the session activities to be enjoyable, relevant and interesting; and the skills they learnt to be useful. The values instilled in the group, of respect and autonomy, and the people and relationships were reported to be central positive elements to the sessions, as was the variety of creative activities. Some young people noted that they would have liked more opportunities for ‘serious’ discussion, whether this be mental health or otherwise.

#### Semi‐structured focus group interviews

A total of 25 of the 28 young people who completed the programme took part in the focus group interviews. We were unable to complete the intended qualitative feedback session with year 1 parents/carers due to COVID‐19 disruptions. Four of the second‐year parents attended an online focus group at the end of the programme and a further eight parents/carers completed self‐report written versions of the interview. Parent/carer participants were 83% mothers.

The coding matrix derived from the framework analysis of participant and parent/career feedback interviews, with example responses, is shown in Table 3.

**TABLE 3:** Thematic codes and descriptive examples from focus groups with young people and carers.

Category	Coded themes	Descriptors	Example responses
Intervention Design	Unconventional therapy	Novel Variety Psychology + Arts	‘it's like therapy, but it's not like therapy, but it is at the same time’ ‘in IF you do skills but we go onto the arts part and then use it which for me is helpful because you can't just feed information to me otherwise it's not going to stick in my head’. ‘different, kind of every time’, ‘refreshing’, ‘adventurous’, ‘exploratory discovery’ ‘it's a good change…like one day arts, one day psychology’ ‘I like how they cross‐over (the arts and psychology)’, ‘it's better blended together’
	Arts as motivating	Creativity Self‐expression	‘I just wanted to go because I heard it was about arts’ ‘the performing arts and art, that was important for me’; ‘Art I think was the best bit’ ‘it allowed her to delve more into creativity and enjoy expressing herself’
	Logistics	Routine Structure Incentives Materials	‘it's given me routine and structure’, ‘I like the structure’, ‘the routine’. ‘the pizza was a big incentive’ ‘I put the booklet on my wall so I can look at it’ ‘some A4s were given and one was stuck to our fridge – STOP ‐ all of those little things you have shared were fundamental’
Intervention Process Intervention Process cont.	Community	Shared experience Belongingness Socializing Trusted facilitators	‘meeting people that are very similar to me or probably would understand what I'm going through was important’ ‘I've created some really good connections with young people and the adults’ ‘I became really close with people, and became friends’ ‘united’ ‘she didn't consider herself the odd one out anymore’ ‘I don't really trust other people…but the members have really changed my perspective on things’ ‘they (facilitators) don't act like teachers’
	Fun	Enjoyment Activation	‘it's been a really good experience’ ‘it was amazing’ ‘It's relatable, like it's something you would enjoy’ ‘she's really enjoyed the programme’, ‘she's so happy on session days’, ‘it does give you something to look forward to’ ‘I think each week she's missed it since it stopped’.

	New skills	DBT skills Arts skills Alternative ways of thinking	‘the PLEASE skills is very helpful, allows me to see why I'm feeling the way I'm feeling’ ‘the learning, new experiences’ ‘I also see she now channels her moments with arts, she coloured her arms with paint, it was a really beautiful way of releasing pent up emotions without harming herself’ ‘I held on to the thing about the triangle which is like your self‐respect, your relationship… what you are prioritizing’ ‘I've used “Check the Facts”’ ‘they have given me more reason to kind of make myself think about it first’ ‘she has equipped herself with more coping mechanisms’
Areas for improvement	Recollection and generalization of skills 1–1 input Amount of content	‘maybe like one to one sessions….with people who like really needed it. I don't know how to explain it. Like, like, just small chats…one‐to‐one’ ‘speaking about issues in a bit more depth’ ‘maybe, more reminders, like a week before, and a day before’ ‘it was a lot like’
Intervention Impact Intervention Impact cont.	Confidence	Confidence Maturity	‘I'm a lot more confident’ ‘it was starting to grow on me like, let's be confident today. I can be a better person today’. ‘she's more confident’ ‘she's got a better character, attitude’
	Managing relationships	Understanding others Communication Making friends	‘a bit more understanding about people’ ‘I think understanding just people’ ‘it's been easier (communication), especially with my friends, family, it's made me realize who I need and who I don't’ ‘she's developed more willingness to make friends’ ‘being social and finding a way to find friends, that was massively helpful for her’
	Managing emotions	Awareness Understanding Coping with negatives More positives	‘I'm a bit more self‐aware…there's always more to your emotions than meets the eye’ ‘some progress in a way about sort of understanding emotions’ ‘it calmed down my anger’, ‘better at managing my anger about people looking at me’ ‘she's dealing with negatives each day better’ ‘she's calmer, more relaxed’ ‘she copes better with sadness’ ‘she came out of the worst time. I see she is more positive’; ‘has become more cheerful’.
	Tolerating Distress	Reduced self‐harm	‘because she's not reverted to anything we didn't want to, we think she's good’, ‘knows how to handle herself when in pain’ ‘I've been clean (of self‐harm) for 15 weeks’

	Parental Perceived support	Safe place Relief Help	‘you guys have really kept X safe’ ‘once a week I was happy because I knew my daughter was there’. ‘I was so relieved because it was a difficult period’ ‘what IF has given and provided both myself and my daughter is a safe place, where she could explore whatever and where I was supported too because you gave her something that I couldn't’ ‘I cannot even say it in words, how important IF has been in these two years. You will never know’.
Adolescent–Parent communication	Openness Engagement/asking for help Getting needs met	‘speaking out when there's a problem’ ‘If something upset her and she cried, she now tells me’ ‘she managed to come and talk to me’ ‘now asks for more hugs’, ‘more engagement’ ‘more willing to share with me her own progress’, ‘able to articulate how she is feeling better’.

Thirteen coded themes were identified to describe patterns in the data from initial descriptors and to structure the framework for analysis. These were grouped into three higher‐order categories reflecting aspects of the ‘intervention design’, ‘intervention process’ or ‘intervention impact’ that were in participants' views relevant to questions regarding their engagement with and experience of IF.

Regarding the intervention design, themes identified included ‘unconventional therapy’, the ‘arts as a motivating factor’ and several positive aspects of the ‘logistics’ of the programme, including the location, the structure and routine, incentives and materials. Factors relating to the process of IF delivery which contributed to its acceptability included that it provided a sense of ‘community’ through shared experiences, belongingness and friendship. Contrary perhaps to the views of typical therapy or mental health interventions, people described the experience as ‘fun’ and ‘enjoyable’. The importance of ‘skills development’ was also noted, including DBT skills, arts skills and developing new ways of thinking and new perspectives. Several themes were additionally identified which gave an indication as to the impact of the programme. These included self and parent‐observed changes in: self‐harming, confidence, understanding of others and communication, self‐awareness and coping with negatives, as well as parent–adolescent communication and seeking support from parents. In addition, a theme regarding areas for improvement was identified and this included requests for between‐session contact to support skills use, as well as the potential for 1–1 sessions.

### Secondary quantitative outcomes

Secondary quantitative data were collected at the start and the end of the programme for 26 of the 28 programme completers.

#### Self‐harm

Examination of reported self‐harming data indicated reductions in the frequency of self‐harming behaviour when comparing the 6‐month period prior to programme start, with the 6 months prior to programme end, although these did not reach statistical significance, Fisher's exact test, d.f. = 4, *p* = .20; see Table 4. Two individuals who joined modules 2 and 3 of the second year, after programme set up and distress tolerance skills were excluded from this analysis.

**TABLE 4:** Frequency of self‐harming in preceding 6 months, at start and end of programme.

	Start of programme		End of programme	
	*N*	%	*N*	%
Never	3	12.0	7	29.2
1	2	8.0	2	8.3
2–5	8	32.0	11	45.8
6–10	3	12.0	1	4.2
>11	9	36.0	3	12.5
Total	25	100	25	100

#### Service use

Data collected at baseline on use of clinical health services indicated that 46% of treatment completers had some contact with CAMHS in the preceding 3 months, 38% had contact with another outpatient mental health support, for example, school nurse or social worker, and 54% had seen a counsellor or psychologist in the past. By the end of the programme, the percentage of the participants using these mental services was either the same or less than at the start. The most common service used both at the start and end of the programme was GP, with 73% of the participants accessing GP services (for any ailment) at the start of the programme and 54% at the end of the programme. Use of regular medication was not common either at the start or the end of the programme.

#### Clinical outcomes

Statistically significant differences were observed in the perceived impact of difficulties and perceived social support from a significant other at the end of the programme by comparison to the start of the programme, see Table 5. There were no other statistically significant differences observed in clinical measures.

#### Opt‐out interviews

A total of 11 of the 26 candidates who either did not start the programme after invitation in the first year or opted out during the programme completed a structured opt‐out interview. Reasons reported for non‐completion included: competing school or leisure commitments, year 1 delay in first contact to workshop launch, being too far away, motivation problems, moving out of Borough for safety, COVID shielding, personal problems, including relationship issues and moving home, and all other mental health issues.

## DISCUSSION

In this pilot evaluation to inform future work, we aimed to assess a novel service incorporating a blended programme of arts and psychology skills for young women with a recent episode of self‐harming. The IF programme aimed to increase access to mental health support, and the results suggest that this aim was partially met. Less than half of participants had prior contact with CAMHS, and a majority of referrals were received via non‐CAMHS referral routes. Participants reflected the diverse demographic of the localities from which we were recruiting (Office for National Statistics, 2023a, 2023b) with a majority identifying as from United Kingdom racially minoritized backgrounds. Given what is known about experiences of racial inequality in relation to Black children and young people in CAMHS (Ayodeji et al., 2021; Holt, 2022), it is reassuring that completion rates and comparisons between completers and non‐completers suggested that IF was an acceptable programme for an ethnically diverse group of young women.

The fact that a proportion of referrals were still received via CAMHS suggests that the programme was perceived by professionals as a viable alternative provision for young people unable to access statutory mental health support for whatever reason. The data also highlighted a need for support among school safeguarding teams, who were the second most frequent referrers to the programme. These main referral uptake routes reflect the challenges of cross‐sector working, as is recommended in the NICE guidelines for self‐harm (NICE, September, 2022), in that training and service gaps can emerge at the boundaries for certain issues, where the social and health determinants of problems are unclear. Indeed, the recent evaluation of the national programme which funded the creation of mental health support teams (MHSTs) in schools, stated these challenges, concluding that certain MHST approaches were unsuitable for children with specialist needs and there remained cultural and language barriers to engagement (Ellins et al., 2023). IF represents a prototype model for novel interdisciplinary and inter‐sector working which may meet the well‐being needs of young people perceived to fall between educational and health services (Ellins et al., 2023).

Given the length and complexity of the group, completion rates were promising and within range for community DBT interventions (24%–58%, Landes et al., 2016). Our qualitative results suggested many aspects of the service performed to support engagement and the experience of participants. The arts elements of the group, including the facilities and range of activities, were a crucial factor in supporting engagement and programme completion. Participant relationships with the practitioners were also a key strength, possibly influenced by the diversity of the facilitators themselves and perceptions of safety associated with a women‐only space. The importance of themes related to social factors, views and attitudes towards the professionals involved is consistent with previous research into overcoming barriers to seeking help among adolescents (Radez et al., 2021). However, several questions remain regarding which parts of the programme were necessary or sufficient to support involvement, and for whom the programme was most fitting. We must not overlook the limited scope of this study in that it did not include young men. Also, that the only two ‘Looked After’ young people did not complete the programme suggests that it may not have met the requirements of young people with care experiences.

Similarly, questions remain regarding the impact of the programme. Quantitative results were reassuring, with significant reductions observed in the perceived impact of difficulties and increase in perceived social support and prosocial behaviours at the end of the programme by comparison to the start. However, the study was underpowered to detect meaningful changes in clinical outcomes and assumptions about effectiveness could not be inferred from the study design. Reductions in the frequency of self‐harming were also observed, however, it was notable that some young people continued to have incidences of self‐harming towards the end of the programme.

Overall, the results of the evaluation might most helpfully be interpreted within the context of developments in ‘social prescribing’ globally. ‘Social prescribing’ seeks to recognize the benefits of community connections, like gardening, arts and cultural activities, volunteering or befriending to people's emotional well‐being and health (Morse et al., 2022). There has been a significant push nationally in the United Kingdom, and further afield, to report the benefits of ‘social prescribing’ and to develop policy to formalize and support programmes in the area and the signposting of people thereto (All Parliamentary Group on Arts Health and Wellbeing, 2023). There remain many criticisms of the approach, including that it represents an eschewing of responsibility within existing health care systems and that the intrusion of medicalized language within cultural activities has a deleterious effect on people's experiences (Gibson et al., 2021). However, the results of this evaluation of ‘Imagining Futures’ are consistent with previous positive reviews of such programmes (Dow et al., 2023; Fancourt & Finn, 2019). The key issues the current study raises, for future research work, have also previously been reported in the literature. Namely, there remain questions about which populations should be prioritized to focus efforts upon (Morse et al., 2022) and what level of research evidence should be sought to demonstrate the effectiveness of programmes (Estevao et al., 2021; Husk et al., 2019). There are also broader ethical issues of interest to interdisciplinary programmes including the safeguarding, training and competency frameworks for practitioners (Calderón‐Larrañaga et al., 2021).

### Limitations

Despite many strengths of the programme and evaluation, there are several limitations. We must note the extraordinary influence of the COVID‐19 pandemic and its impact on the interpretability of the results. While the pandemic presented many challenges, it is possible that there was positive bias to people's views of the programme, given the perceived absence of other services and supports at this time. However, the programme was delivered both pre‐ and post‐COVID‐19, incorporating feedback from participants. Its continued success, including improved completion rates in the second year despite the challenges of COVID‐19, is a testament to its feasibility. Given the central aim of improving access, it is notable that there was no measure of deprivation or socio‐economic status. However, we were also trying to minimize the measurement burden and association with services and research for participants. The measure of self‐harm was rudimentary and only allowed a comparison between 6 months prior to the programme and the latter 6 months at the end of the programme (Table 4). It is possible therefore that we may have underestimated improvements in self‐harming behaviour. The formative nature of the evaluation means that only tentative suggestions can be made about the influential and ‘active ingredients’ of the programme. However, we have addressed this by the inclusion of rich qualitative information derived directly from participants, which has provided avenues for future research and development. As mentioned previously, the absence of data relating to those who opted out of the programme has meant we were unable to complete ITT analyses which might increase the likelihood of Type 2 errors in our quantitative results. However, we have already observed caution in interpreting these results as it is likely we were underpowered to detect meaningful changes. Notably, given the importance of the artistic elements to programme participants, we are also limited here in our ability to reflect and acknowledge the creative processes and outputs. We are also limited in the extent to which we can comment on the active ingredients of the programme, be these DBT or art elements.

**TABLE 5:** Clinical Outcomes at start and end of programme—completers only.

	*N*	Start $\bar{X}\;\left(s.d\right)$	End $\bar{X}\;\left(s.d\right)$	Estimated diff. (95%CI)	*p* Value
SDQ					
Emotional	26	6.81 (2.14)	6.31 (2.19)	.50 (−.31 to 1.31)	.22
Conduct	26	3.81 (1.63)	3.50 (1.90)	.31 (−.35 to .96)	.34
Hyperactivity	26	5.85 (1.59)	6.00 (1.39)	−.15 (−.77 to .46)	.62
Peer problems	26	5.00 (1.20)	5.19 (1.10)	−.19 (−.74 to .36)	.48
Prosocial	26	7.15 (1.80)	7.65 (1.74)	−.50 (−.1.04 to .04)	.07
Total difficulties	26	21.46 (3.80)	21.00 (4.10)	.46 (−.96 to 1.88)	.51
**Impact**	**25**	**4.56 (2.31)**	**3.20 (2.31)**	**1.36 (.33 to 2.39)**	.**01**
DERS					
Strategies	26	11.62 (2.77)	11.08 (3.45)	0.54 (−0.83 to 1.91)	0.43
Non‐acceptance	26	9.85 (3.33)	9.53 (3.93)	0.31 (−1.06 to 1.68)	0.65
Impulse	26	10.38 (3.43)	9.23 (3.98)	1.16 (−0.36 to 2.67)	0.13
Goal‐directed	26	12.81 (2.39)	12.00 (3.20)	0.81 (−0.45 to 2.06)	0.20
Awareness	26	9.15 (3.02)	9.58 (2.83)	−0.42 (−1.36 to 0.51)	0.36
Clarity	26	9.15 (2.80)	8.19 (3.58)	0.96 (−0.33 to 2.26)	0.14
WWB Total	26	36.50 (7.82)	40.85 (2.63)	−4.34 (−9.96 to 1.27)	0.12
PSS					
Total PSS	23	51.74 (14.63)	53.65 (18.27)	−1.91 (−8.07 to 4.25)	0.53
Friends	25	16.72 (6.43)	16.60 (8.32)	0.12 (−2.56 to 2.80)	0.93
**Significant Other**	**24**	**17.67 (6.74)**	**20.33 (7.80)**	**−2.67 (−5.00 to −0.33)**	**0.03**

*Note*: Bold indicates significant at *p* < 0.05 level.

## CONCLUSIONS

The study has demonstrated the significant potential of NHS third‐sector collaborations and psychology–arts fusions. However, many outstanding questions and areas for development remain. Future work might helpfully focus on co‐design with young people to revise and redact the programme towards particular mental health targets and local needs. Future research is needed into the overlapping or additive mechanisms of change in psychology and arts practices, especially given the potential that joint programmes present to reach and resonate with diverse communities.

## AUTHOR CONTRIBUTIONS

**L. M. Smith:** Conceptualization; writing – original draft; methodology; writing – review and editing; formal analysis; project administration; investigation. **B. Barrett:** Conceptualization; methodology; writing – review and editing. **S. Barnes:** Conceptualization; funding acquisition. **B. Oltean:** Data curation; formal analysis. **L. Ige:** Data curation; formal analysis. **C. Day:** Conceptualization; funding acquisition; methodology; supervision. **T. Tranah:** Conceptualization; funding acquisition; writing – review and editing; methodology; project administration; validation; supervision; investigation.

## FUNDING INFORMATION

The study was funded by a grant awarded by the Guy's and St Thomas's Charitable Foundation.

## CONFLICT OF INTEREST STATEMENT

The authors whose names are listed below certify that they have NO affiliations with or involvement in any organisation or entity with any financial interest (such as honoraria, educational grants, participation in speakers' bureaus, membership, employment, consultancies, stock ownership or other equity interest, and expert testimony or patent licensing arrangements), or nonfinancial interest (such as personal or professional relationships, affiliations, knowledge or beliefs) in the subject matter or materials discussed in this manuscript.

## Supporting information


Data S1‐S4

## Data Availability

The dataset is available on request by qualified researchers–scientists. Requests require a concept proposal describing the purpose of data access, appropriate ethical approval and provision for data security. All data analysis scripts and results files are available for review.
