# Supplementary material for: Imagining Futures: Evaluation of a blended programme of dialectical behaviour therapy and the creative arts for young women with a history of self‐harm
Source: Br J Clin Psychol. 2025 Feb 19;64(3):702–24. doi: 10.1111/bjc.12528 (PMC12334986; doi:10.1111/bjc.12528)
Supplement: Supplementary file 1 — Data S1‐S4 [file BJC-64-702-s001.docx]

**
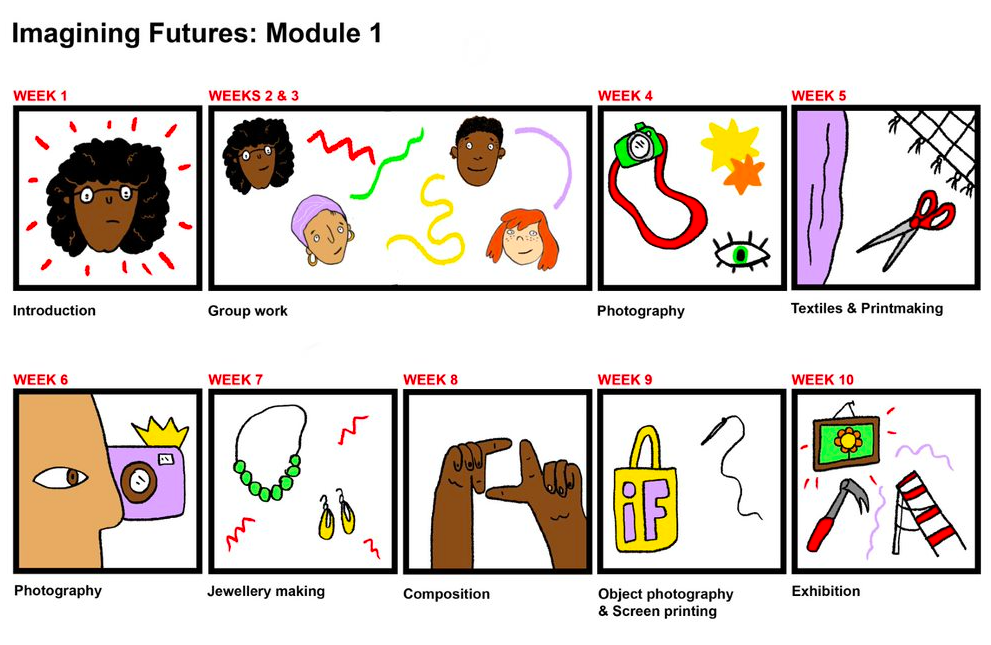
**


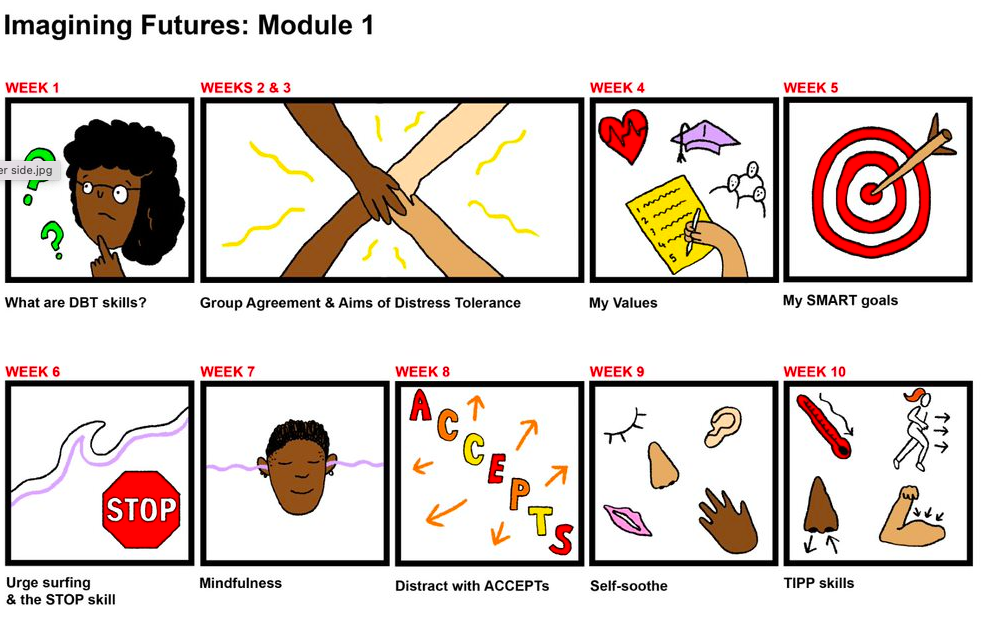


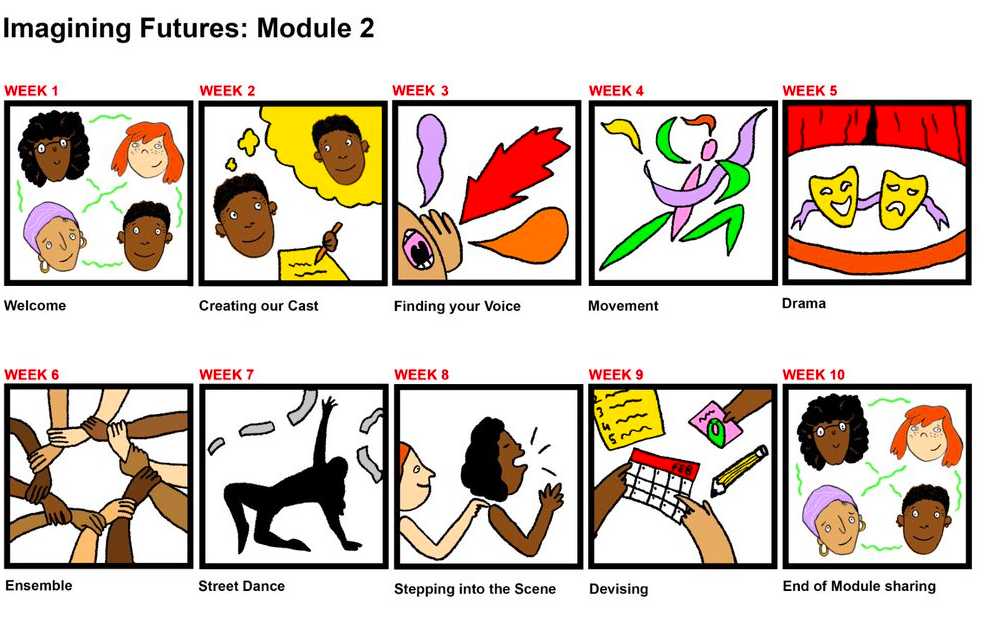


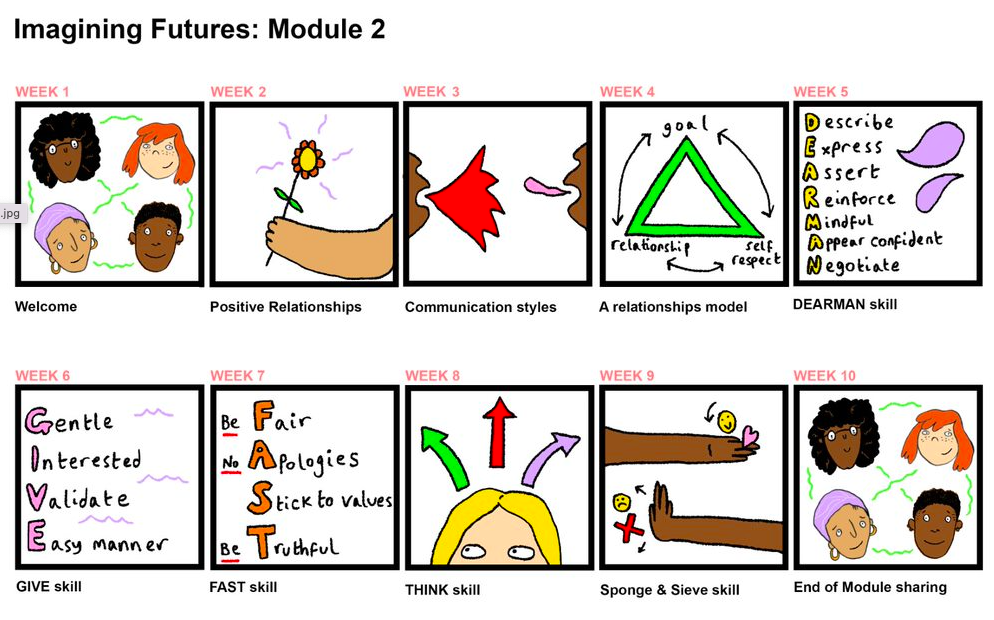


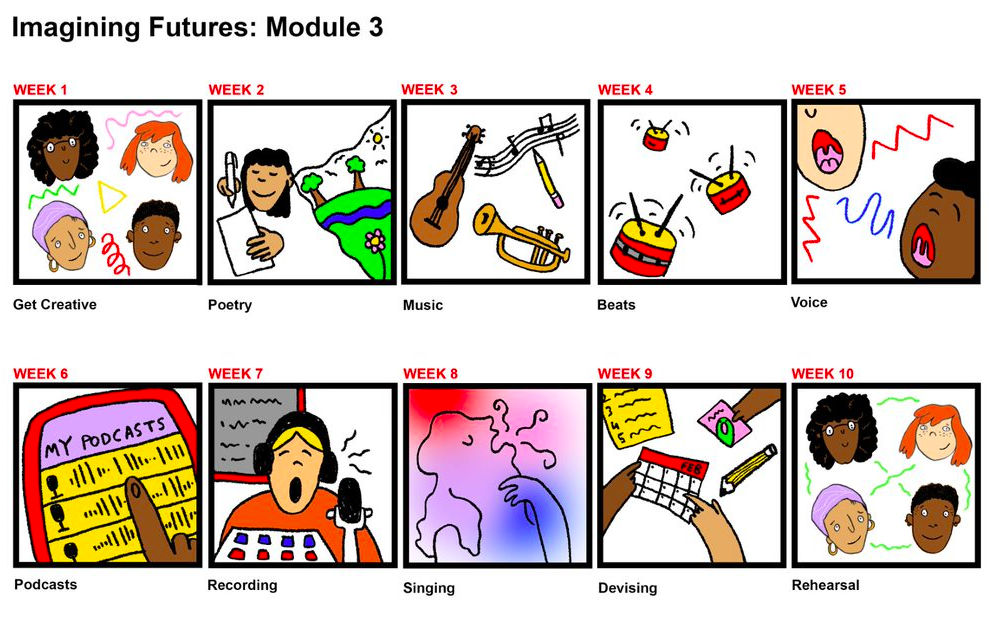


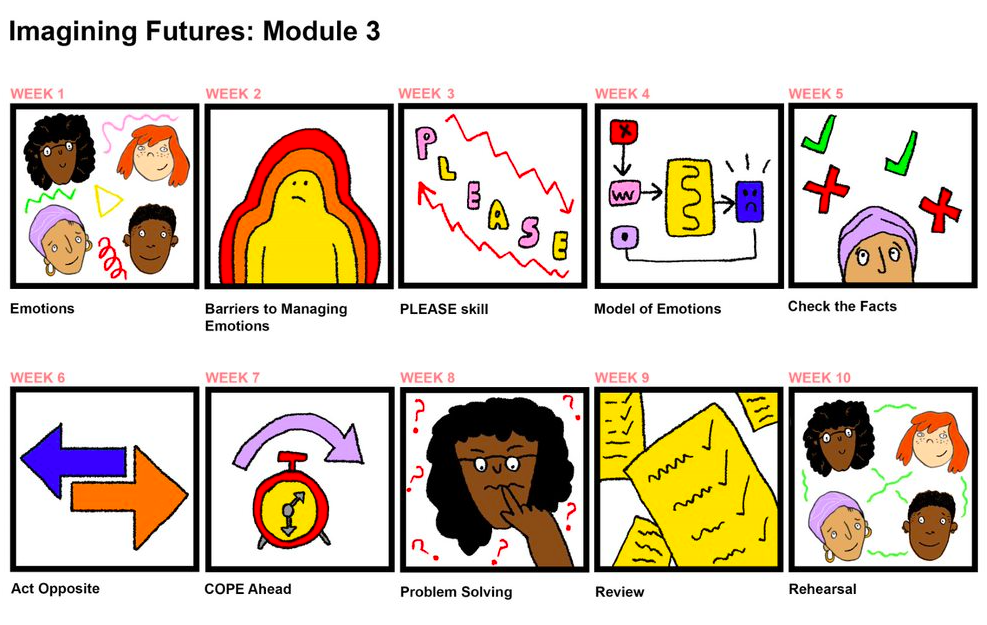


1. **Examples from Skills Resource book**

**
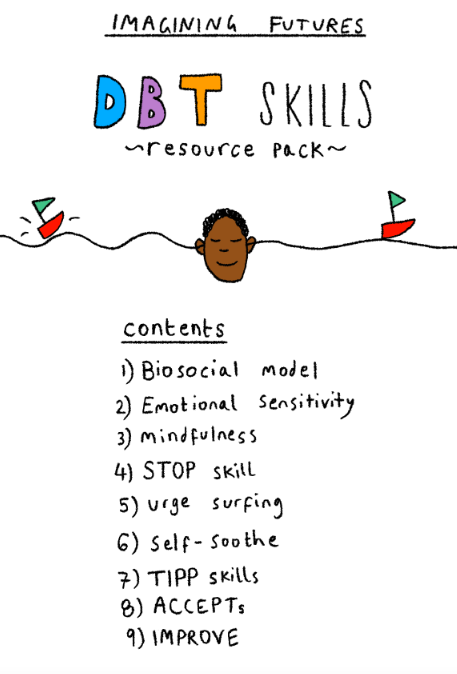
**

**
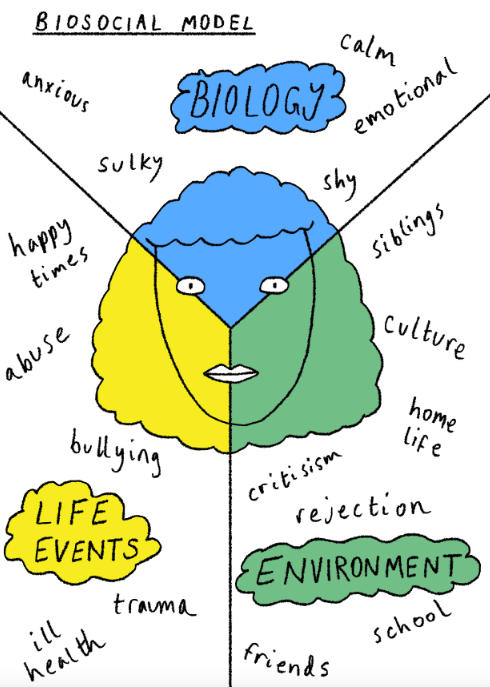
**

**
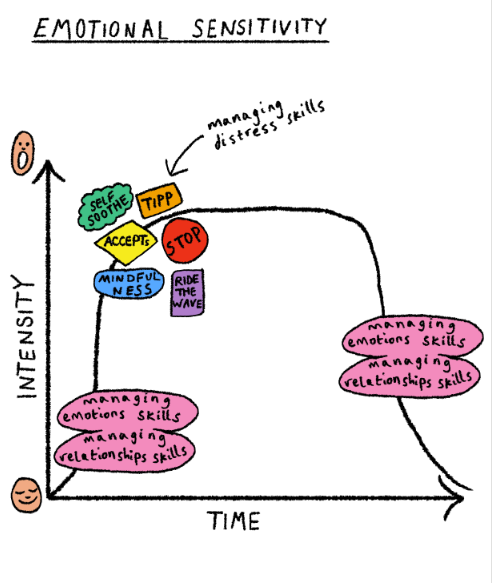
**

1. **Semi-structured interview schedules**

**Young people:**

*Access/Engagement questions:*

1. Tell us about your experience of hearing about the group, and your decision to come.
2. Can you recommend any other ways in which to reach out directly to young people e.g. social media.
3. What has kept you coming to the group?

What has your experience been of the Imagining Futures team (facilitators and other young people)?

*Acceptability/Impact questions:*

1. What one word would you use about the group?
2. What was life like before you started the workshops? Have things remained the same or changed over the time you were attending the workshops?
3. Has the course had any impact on your response to difficult situations?
4. To what extent did you like the way the group was delivered?
5. Do you feel the workshops helped you achieve your arts and wellbeing goals?

**Parents:**

*Access/Engagement questions*

1. What was it about the programme that initially attracted your child/you to Imagining Futures?
2. Was there anything further that we could have communicated or provided at the project outset to support your child/you better?
3. What kept your child coming to the group?
4. Are there any specific ways that you have had to support your child to attend? e.g. reminders, providing transport, problem solving between sessions.

What has your experience been of the Imagining Futures team (facilitators and other young people)?

1. Did you attend any of our parent evenings? Was there anything further we could have done to have supported your involvement?

*Acceptability/Impact questions:*

1. In what ways has the group affected your and your child’s life?
2. What changes, if any, have you noticed in your child’s behaviour or how they spend their time?
3. Has the course had any impact on your child’s response to difficult thoughts, feelings or situations? (in what ways)
4. Has the course had any impact on your child’s enthusiasm or engagement in the arts and creativity?

# Table 1: Comparison in baseline clinical data: completers vs non-completers

|  | N | Completers  $\overline{X} (s.d)$ | Non-completers  $\overline{X} (s.d)$ | Estimated diff. (95%CI) | P Value |
| --- | --- | --- | --- | --- | --- |
| N | 42 | 27 | 15 | - | - |
| McLean Screen Total |  | 7.81 (1.49) | 8.07 (1.03) | -0.25 (-1.13 – 0.63) | 0.11 |
|  |  |  |  |  |  |
| SDQ |  |  |  |  |  |
| **Emotional** |  | **6.78 (2.10)** | **6.87 (1.06)** | **-0.09 (-1.26 – 1.09)** | **0.05** |
| Conduct |  | 3.78 (1.60) | 4.00 (1.55) | -0.22 (0.51 **–** -1.25) | 0.69 |
| Hyperactivity |  | 5.81 (1.57) | 5.67 (1.45) | 0.15 (0.49 **–** -0.85) | 0.92 |
| Peer problems |  | 4.93 (1.23) | 5.07 (1.16) | -0.14 (0.39 **–** -0.93) | 0.47 |
| Prosocial |  | 7.19 (1.78) | 6.93 (1.94) | 0.25 (0.59**–** -0.94) | 0.50 |
| Total difficulties |  | 21.30 (3.83) | 21.60 (3.56) | -.30 (1.20 **–** -2.74) | 0.60 |
| Impact |  | 4.56 (2.22) | 5.27 (2.22) | -0.71 (0.72 **–** - 2.16) | 0.75) |
| DERS |  |  |  |  |  |
| Strategies |  | 11.48 (2.80) | 11.60 (2.99) | -0.12 (0.93 **–** -1.99) | 0.66) |
| Non-acceptance |  | 9.67 (3.40) | 9.73 (3.22) | -0.67 (1.07 **–** -2.24) | 0.78 |
| Impulse |  | 10.33 (3.37) | 11.27 (4.27) | -0.93 (1.20 **–** -3.35) | 0.18 |
| Goal-directed |  | 12.89 (2.38) | 12.93 (2.31) | -0.04 (0.76 **–** -1.58) | 0.46 |
| Awareness |  | 9.07 (2.98) | 8.60 (3.22) | 0.47 ( 0.99 **– -**1.53) | 0.64 |
| Clarity |  | 9.04 (2.81) | 8.60 (3.14) | -1.96 (0.94 **–** -3.87) | 0.90 |
| DERS Total |  | 62.49 (2.04) | 65.13 (2.97) | 2.65 (3.52 **–** -9.76) | 0.79 |
| WWB Total |  | 36.56 (7.68) | 32.73 (11.86) | -3.82 (3.01 – - 2.27) | 0.10 |
| PSS |  |  |  |  |  |
| Total PSS |  | 51.40 (14.12) | 53.80 (15.55) | -2.40 (4.79**–** -12.09) | 0.44 |
| Friends |  | 16.56 (6.20) | 18.67 (6.48) | -2.11 (2.03 **–** -6.21) | 0.78 |
| Significant Other |  | 17.92 (6.70) | 19.53 (6.81) | -1.61 (2.21 **–** -6.14) | 0.74 |
|  |  |  |  |  |  |

**Table 2: Comparison in self-report risk information obtained at application**

| Category | ExperEExperience | Frequency Completers (%) | Frequency Non Completers (%) |
| --- | --- | --- | --- |
| N |  | 28 | 17 |
|  |  |  |  |
| Emotional Health | Low Self-Esteem | 20 (87) | 11 (79) |
|  | Mood Swings | 16 (70) | 10 (71) |
|  | Irritation/Anger | 15 (65) | 9 (64) |
|  | Low Mood | 19 (83) | 14 (100) |
|  | Suicidal Thoughts | 18 (78) | 10 (71) |
|  | Anxiety | 15 (57) | 11 (79) |
|  |  |  |  |
| Social/Environmental | Family Difficulties | 13 (57) | 13 (93) |
|  | Relationship Difficulties | 10 (44) | 6 (43) |
|  | Social Isolation | 8 (35) | 7 (50) |
|  | Housing/unsuitable housing | 4 (17) | 3 (21) |
|  | Bullying | 7 (30) | 2 (14) |
|  |  |  |  |
| Risk of Harm | Periods of going missing | 0 | 1 (7) |
|  | Have harmed others | 0 | 2 (14) |
|  | Gang association/involvement | 0 | 1 (7) |
|  | Abusive/exploitative sex | 4 (17) | 0 |
|  | Commercial sex | 1 (4) | 1 (7) |
|  |  |  |  |
| Problem behaviours | Self-harming | 19 (83) | 9 (64) |
|  | Thoughts/urges to self-harm | 17 (74) | 12 (86) |
|  | Other problem behaviours | 3 (13) | 2 (14) |
|  | Eating Issues | 7 (30) | 5 (36) |
|  | Substance misuse | 3 (13) | 2 (14) |
|  | Involvement in criminal justice system | 0 | 1 (7) |
|  |  |  |  |
|  | Missing | 5 (19) | 3 (17) |
